# Antiallodynic effect of PhAR‐DBH‐Me involves cannabinoid and TRPV1 receptors

**DOI:** 10.1002/prp2.663

**Published:** 2020-09-23

**Authors:** Geovanna Nallely Quiñonez‐Bastidas, Oscar Palomino‐Hernández, Manuel López‐Ortíz, Héctor Isaac Rocha‐González, Gloria Melisa González‐Anduaga, Ignacio Regla, Andrés Navarrete

**Affiliations:** ^1^ Facultad de Química Departamento de Farmacia Universidad Nacional Autónoma de México Ciudad Universitaria Coyoacán Ciudad de México México; ^2^ Computational Biomedicine ‐ Institute for Advanced Simulation (IAS‐5) and Institute of Neuroscience and Medicine (INM‐9) Forschungszentrum Jülich Jülich Germany; ^3^ Department of Chemistry Rheinisch‐Westfälische Technische Hochschule Aachen Aachen Germany; ^4^ Facultad de Estudios Superiores Zaragoza Universidad Nacional Autónoma de México (UNAM) México DF México; ^5^ Sección de Estudios de Posgrado e Investigación Escuela Superior de Medicina Instituto Politécnico Nacional México Ciudad de México México

## Abstract

The antiallodynic effect of PhAR‐DBH‐Me was evaluated on two models of neuropathic pain, and the potential roles of CB1, CB2, and TRPV1 receptors as molecular targets of PhAR‐DBH‐Me were studied. Female Wistar rats were submitted to L5/L6 spinal nerve ligation (SNL) or repeated doses of cisplatin (0.1 mg/kg, i.p.) to induce experimental neuropathy. Then, tactile allodynia was determined, and animals were treated with logarithmic doses of PhAR‐DBH‐Me (3.2‐100 mg/kg, i.p.). To evaluate the mechanism of action of PhAR‐DBH‐Me, in silico studies using crystallized structures of CB1, CB2, and TRPV1 receptors were performed. To corroborate the computational insights, animals were intraperitoneally administrated with antagonists for CB1 (AM‐251, 3 mg/kg), CB2 (AM‐630, 1 mg/kg), and TRPV1 receptors (capsazepine, 3 mg/kg), 15 min before to PhAR‐DBH‐Me (100 mg/kg) administration. Vagal stimulation evoked on striated muscle contraction in esophagus, was used to elicited pharmacological response of PhAR‐DBH‐ME on nervous tissue. Systemic administration of PhAR‐DBH‐Me reduced the SNL‐ and cisplatin‐induced allodynia. Docking studies suggested that PhAR‐DBH‐Me acts as an agonist for CB1, CB2, and TRPV1 receptors, with similar affinity to the endogenous ligand anandamide. Moreover antiallodynic effect of PhAR‐DBH‐Me was partially prevented by administration of AM‐251 and AM‐630, and completely prevented by capsazepine. Finally, PhAR‐DBH‐Me decreased the vagally evoked electrical response in esophagus rat. Taken together, results indicate that PhAR‐DBH‐Me induces an antiallodynic effect through partial activation of CB1 and CB2 receptors, as well as desensitization of TRPV1 receptors. Data also shed light on the novel vanilloid nature of the synthetic compound PhAR‐DBH‐Me.

## 1 INTRODUCTION

Neuropathic pain is defined as a pain caused by a lesion or disease of the somatosensory nervous system^1^, which produces functional disabling symptoms and anxiety disorders, addressing on the quality of life of those who suffer this condition^2^. Furthermore, pharmacological treatment of neuropathic pain has limited efficacy and provides an unsatisfactory relief, most often accompanied by side effects which complete an aberrant process that impairs the management and prognosis of the patients^3^, ^4^, ^5^. In a recent report, the Canadian Pain Society published a consensus statement for pharmacological management of chronic neuropathic pain, which includes cannabinoids as a third‐line of treatment^6^. Thus, current trends in pharmacological research for neuropathic pain management consider the development of cannabinoid‐like drugs and the subsequent characterization of its molecular mechanism of action^7^.

Cannabinoid drugs exert their anti‐neuropathic effects through activation of CB1 and CB2 receptors at peripheral^8^, ^9^, central^10^, ^11^ and supraspinal levels^12^. Interestingly, alternative targets have also been linked for the treatment of neuropathic pain. Some studies demonstrate that TRPV1 has a special role on antineuropathic‐like behavior induced by anandamide^10^, ^13^.

From a historical perspective, cannabinoid‐based therapy has been used to treat pain on many cultures. In a previous study, we developed the new synthetic compound (*R,Z*)‐18‐((1*S*,4*S*)‐5‐methyl‐2,5‐diazabicyclo[2.2.1]heptan‐2‐yl)‐18‐oxooctadec‐9‐en‐7‐ylphenylacetate (PhAR‐DBH‐Me), which is a diazabicyclic amide synthesized from phenylacetylricinoleic acid and (1*S*,4*S*)‐2,5‐diazabicyclo[2.2.1]heptane. On the first assays, PhAR‐DBH‐Me produced an antinociceptive effect by activation of the CB1 receptor, suggesting a cannabinergic nature for the compound, similar to anandamide^14^. However, further studies on the nature of this compound such as its antiallodynic effect or its participation on CB2 and TRPV1 receptors have not been performed yet.

As anandamide produces antineuropathic effects through activation of CB1, CB2, and TRPV1 receptors and has structural similarity with PhAR‐DBH‐Me, in this work, it was hypothesized that PhAR‐DBH‐Me has antiallodynic effect on SNL‐ and cisplatin‐induced neuropathy and that such effect may be also linked to the activation of the CB1, CB2, and TRPV1 receptors.

## 2 MATERIAL AND METHODS

### 2.1 In vivo assays

#### 2.1.1 Animals

Female Wistar rats (160‐180 g) were used to induce experimental allodynia. Animals were acquired from Centro UNAM‐Envigo (Envigo México, SA de CV) and maintained at controlled room temperature in a 12 h light/dark cycle with food and water ad libitum. In this study, the number of animals used per group was the minimum to obtain statistical significance. Animals were euthanized at the end of experiment. The animals used in this work were handled following the Guidelines on Ethical Standards for Investigation of Experimental Pain in Animals^15^, with the requirements published by SAGARPA in the Technical Specifications for the Production, Care and Use of Laboratory Animals (NOM‐062‐ZOO‐1999), and in a compliance with international rules as the Guide for the Care and Use of Laboratory Animals (National Research Council). In addition, the study was approved by the Ethics Committee for the Use of Animals in Pharmacological and Toxicological Testing (Faculty of Chemistry, UNAM) with the code OFICIO/FQ/CICUAL/355/19.

#### 2.1.2 Drugs

PhAR‐DBH‐Me was synthetized at Facultad de Estudios Superiores de Zaragoza ‐ UNAM, as was described by^14^. Capsazepine was acquired from Tocris Bioscience (Ellisville, MO, USA), whereas AM‐251 and AM‐630 were acquired from Sigma Aldrich (San Luis, MO, USA). Cisplatin was used from a commercial presentation (PISA Laboratories, Mexico), at a concentration of 1 g/ml. Capsaicin was obtained from Sigma‐Aldrich (St. Louis Mo. USA). Sodium pentobarbital was acquired from PISA laboratories.

#### 2.1.3 L5/L6 Spinal nerve ligation‐induced neuropathy

Allodynia was induced in the rats by Kim and Chung surgery^16^. Animals were anesthetized with a mixture of ketamine/xylazine (45/12 mg/kg, i.p.). After surgical preparation of the dorsal vertebral column, the left L5 and L6 spinal nerves were exposed and tightly ligated with 6‐0 silk suture distal to the dorsal root ganglion. For sham‐operated rats, the nerves were exposed but not ligated. The incisions were sutured, and the animals were observed until their recovery. Fourteen days after surgery, allodynia was evaluated.

#### 2.1.4 Cisplatin induced‐neuropathy

In order to induce neuropathy, animals were exposed to repeated intraperitoneal injections of cisplatin (0.1 mg/kg) for 15 days, every third day. To prevent nephrotoxicity, the cisplatin‐treated rats received a second intraperitoneal injection containing saline solution at 0.9% (2 ml/kg). Allodynia was evaluated 15 days after the first administration of cisplatin^17^, ^18^.

#### 2.1.5 Allodynia assessment

Tactile allodynia was determined by the up‐down method^19^. Rats exhibiting motor deficiency were discarded from tactile allodynia evaluation. Tactile allodynia was determined measuring paw withdrawal in response to probing with a series of calibrated fine filaments (von Frey filaments) ranging from 0.4 to 15 g. The stimulus intensity required to produce a response in 50% of the applications for each animal was defined as the 50% withdrawal threshold. All rats were verified for allodynia before experiment (responding to a stimulus of less than 4 g). The 50% of withdrawal threshold of the paw rat, was evaluated in a temporal course of 8 h. For all experiments a blind design was used, drugs were administrated for other personal, avoiding that experimenter associate the nociceptive behavior to the employed treatment. The area under the curve (AUC) was constructed from the temporal course, using the trapezoidal method. Then, from the AUC of the groups, we calculated the percentage of maximum possible effect (%MPE), using the following equation:

$$\text{\% MPE =}\;\;\frac{{\text{AUC}}_{\text{Compound}}\;‐\;{\text{AUC}}_{\text{Vehicle}}}{{\text{AUC}}_{\text{Sham}}\;‐\;{\text{AUC}}_{\text{Vehicle}}}\;x\;100$$

### 2.2 In silico studies

Crystallized structures of the CB1, CB2, and TRPV1 receptors were retrieved from the Protein Data Bank^20^ (PDB codes 5XR8^21^, 5ZTY^22^, and 5IRZ^23^, respectively).

The structures were manually curated using Maestro 12.1 with the Protein Preparation Wizard from the Schrodinger suite 2019‐4^24^. After removal of unnecessary molecular entities in each structure, the hydrogen‐bond network and rotameric conformations were optimized and a restrained minimization was performed. All docking procedures were performed with Glide 8.4 with the SP methodology^25^ and the OPLS3e forcefield^26^.

### 2.3 Vagal nerve stimulation induced esophagus contractions: isolated organ assay

For isolated organ bioassay, a Krebs‐Henseleit modified solution (KHS, mM: NaCl 136.9, KCl 2.7, CaCl_2_ 1.8, MgCl_2_ 2.1, NaH_2_PO_4_ 0.4, NaHCO_3_ 1.9 y glucosa 5.5) was freshly prepared before experiment. Firstly, animals were administrated with an overdose of sodium pentobarbital injection (150 mg/kg, i.p.) and then, a segment of the middle thoracic esophagus (a 1 cm‐long) was dissected and immediately placed into the Krebs‐Henseleit solution at room temperature. Quickly, the two enclosed vagal nerves were identified and separated from the esophagus striated muscle. In order to record the mechanical activity on esophagus muscle, an organ bath (capacity 60 ml) filled with Krebs solution (pH 7.4) in a continuous bubbling with carbogen gas (95% O_2_ and 5% CO_2_) and constant temperature (35°C) was used. Later, esophagus segment was placed between two rings of nichrome, which were connected as follow, one ring was tied to the bottom of chamber, and the other ring to an isometric force transducer (Grass FT 03E), both rings were suspended and tied using a silk thread. Isometric response was filtered and amplified through an amplifier and recorded in the Acqknowledge software, MP100 version 3.5.3 (Biopack systems, inc). A initial tension of 1.0 g was used to perform the assay, the isolated organ preparation was equilibrated and stabilized during 20 min. After this time, the vagus nerve segment was situated on the top of the electrode located on the isolated organ bath and, an electrical stimulation was applied as square‐wave pulses in intensity of 80 V in a duration of 0.5 ms at intervals of 1 s. The contractile responses were registered during 10 min and, they were taken as control. In order to test the effect of the capsazepine, capsaicin or PhAR‐DBH‐ME, alone and combined, were added 10 µl of the capsazepine (10 mM), capsaicin (0.1 mM) and/or PhAR‐DBH‐Me (100 mM) drugs on the same nerve segment. Each vagal nerve was used only one time for each experiment.

Finally, data were processed from the area under the curve (AUC) of the temporal course of contractile response, which was calculated as follows:

%*E* Drug = (compound *gf* × 100%*E*)/ (vehicle or control *gf*).

Were, contractile effect was measured as grams force (*gf*), observed as the spike height in the polygraph.

### 2.4 Experimental design

To determinate the antiallodynic effect of PhAR‐DBH‐Me, neuropathic rats received an intraperitoneal administration of vehicle (saline solution with 10% of Tween 20) or increased logarithmic doses of PhAR‐DBH‐Me (3.2, 10, 32, and 100 mg/kg). Pregabalin was used as positive control (10 mg/kg).

In order to understand the possible mechanism of action involved on antiallodynic effect of PhAR‐DBH‐Me, in silico studies were initially performed to characterize the nature of the compound. Molecular docking was done on structures of the CB1, CB2, and TRPV1 receptors.

Once this characterization was finished, in vivo assays were carried out using a series of pharmacological tools. Rats were administrated with the antagonist of TRPV1 capsazepine at 3 mg/kg, i.p.^27^, the selective antagonist of CB1 receptor AM‐251 at 3 mg/kg, i.p.^27^, ^28^ or the selective antagonist of CB2 receptor AM‐630 at 1 mg/kg, i.p)^29^. All antagonists were administrated 15 minutes before systemic administration of PhAR‐DBH‐Me (100 mg/kg, i.p.).

### 2.5 Statistical analysis

In vivo assays show the mean ± standard error of the mean (SEM) of 6 animals, on independent groups for each experiment. The temporal courses were constructed plotting the 50% withdrawal threshold vs time. The dose‐response curve was constructed using the area under the curve obtained through the trapezoidal method. Data were expressed as the percentage of maximum possible effect (%MPE). Statistical differences were determined by one‐way analysis of variance (ANOVA), followed by Tukey's test with a *P* ≤ .05.

## 3 RESULTS

### 3.1 Systemic administration of PhAR‐DBH‐Me produces an antiallodynic effect on SNL‐ and cisplatin‐induced neuropathy in rats

**FIGURE 1:**
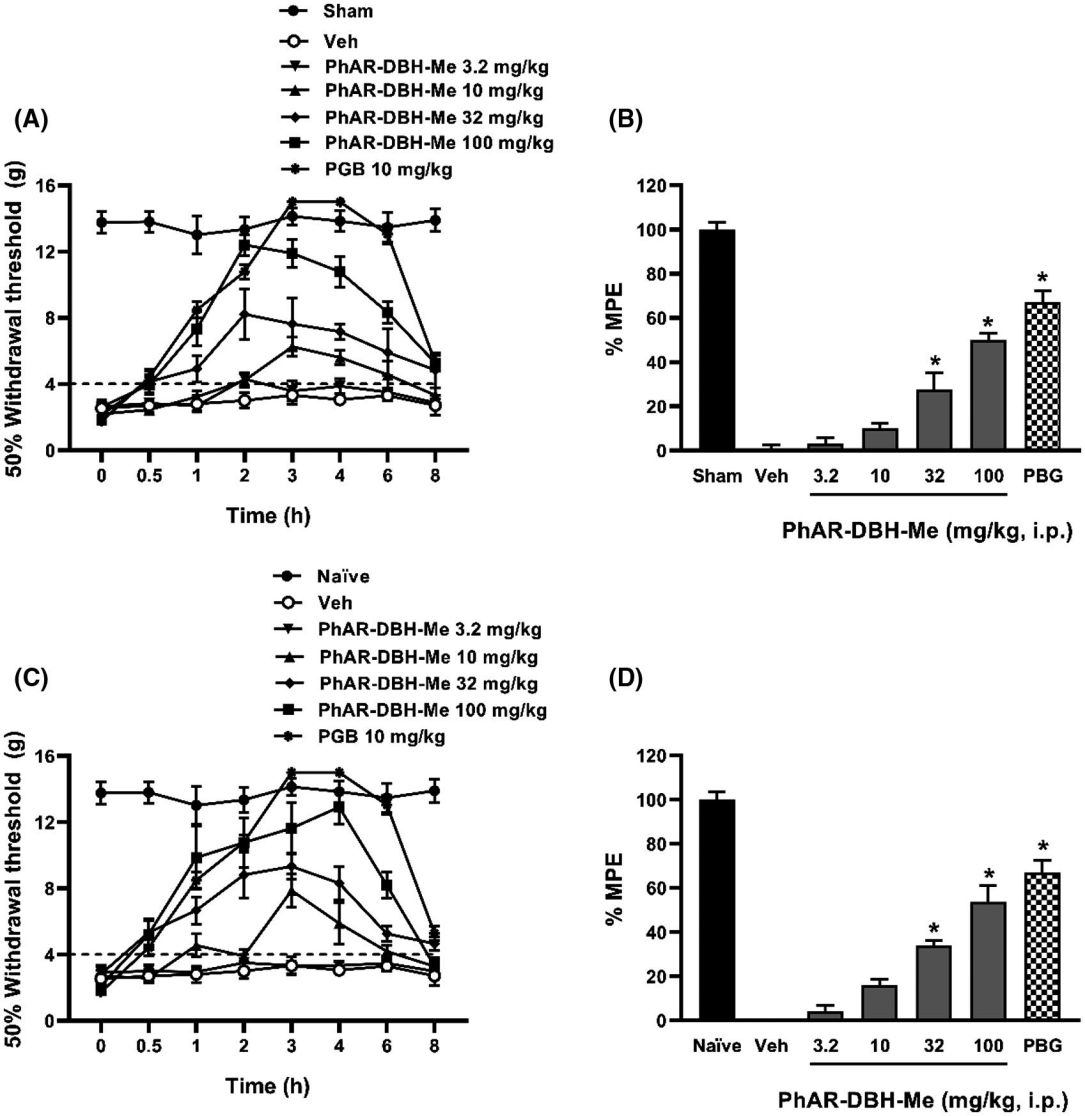
Temporal course of the antiallodynic effect of PhAR‐DBH‐Me in neuropathic rats induced by SNL surgery (**A**) or cisplatin (**C**). Dose‐response curve of the % Maximum Possible Effect (MPE) induced by logarithmic increased doses of PhAR‐DBH‐Me (3.2‐100 mg/kg, i.p.) on allodynia induced by SNL **(B)** or cisplatin **(D)**. Pregabalin (PGB) was used as a reference drug. Data show the mean of 6 animals ± SEM *P < *.0001 vs vehicle was determinate by one‐way analysis of variance (ANOVA), followed by Tukey´s test

The L5/L6 SNL surgery, but not the false ligature, produced allodynia (Figure 1A). Likewise, the repeated intraperitoneal injection of cisplatin (0.1 mg/kg, every third day) produced a drastic reduction in the 50% withdrawal threshold, compared with animals administrated only with the vehicle (Figure 1C). In both models, allodynia was measured 14 days after the L5/L6 SNL surgery or the first cisplatin injection. Moreover systemic administration of increased doses of PhAR‐DBH‐Me (3.2‐100 mg/kg, i.p.) decreased tactile allodynia on L5/L6 spinal nerve ligated rats, as well as cisplatin‐administered rats (Figure 1A and C). PhAR‐DBH‐Me showed an antiallodynic effect in a dose‐dependent manner, which was statistically different (*P* ≤ .001) for 32 and 100 mg/kg in both models. The percentage of maximum possible effect (%MPE) reached was 50.09 ± 2.85% and 53.73 ± 7.43% at 100 mg/kg for SNL and cisplatin model, respectively (Figure 1B and D).

In both experimental models of neuropathy, PhAR‐DBH‐Me administration produced a long‐lasting antiallodynic effect, being observed since the first hour from administration to the eighth hour. Thus, data suggest that the synthetic compound PhAR‐DBH‐Me has an antiallodynic effect on experimental neuropathy induced by either SNL or cisplatin in rats.

### 3.2 PhAR‐DBH‐Me interacts with the binding pocket of CB1, CB2, and TRPV1 receptors

Once evaluated the antiallodynic effect of the PhAR‐DBH‐Me, in silico studies were performed. As baseline compounds with known activity, the antagonists AM251, AM630, and capsazepine (CPZ), as well as, the endogenous agonist anandamide (AEA) were considered.

**FIGURE 2:**
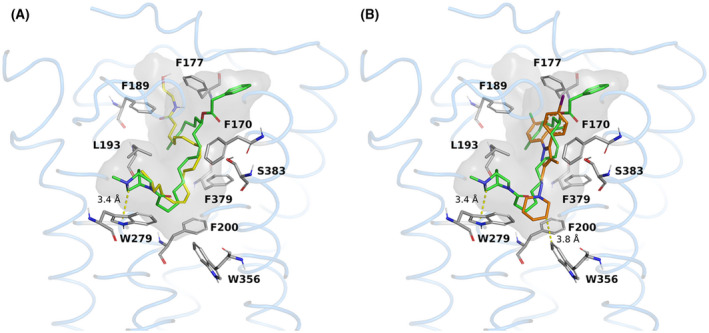
Binding mode comparison between PhAR‐DBH‐Me (green) and the endogenous agonist anandamide (yellow) **(A),** or the synthetic antagonist of CB1 receptor, AM‐251 (orange) **(B),** in the binding pocket of CB1 receptor. The binding cavity is shown in grey, and relevant residues are included

Regarding the CB1 receptor, docking studies show similar binding poses between PhAR‐DBH‐Me and AEA (Figure 2A). The docking scores are −8.05 and −7.8 kcal/mol, respectively. The close scoring values suggest that PhAR‐DBH‐Me is a likely binder to CB1, as the docking score of it is similar to a known agonist. In contrast, the antagonist AM251 shows a different binding pose, where it can be seen hindering the movement of residues F200/W356, important for agonist effects (Figure 2B). The docking score of AM251 is slightly larger than the one for PhAR‐DBH‐Me (−9.9 kcal/mol).

**FIGURE 3:**
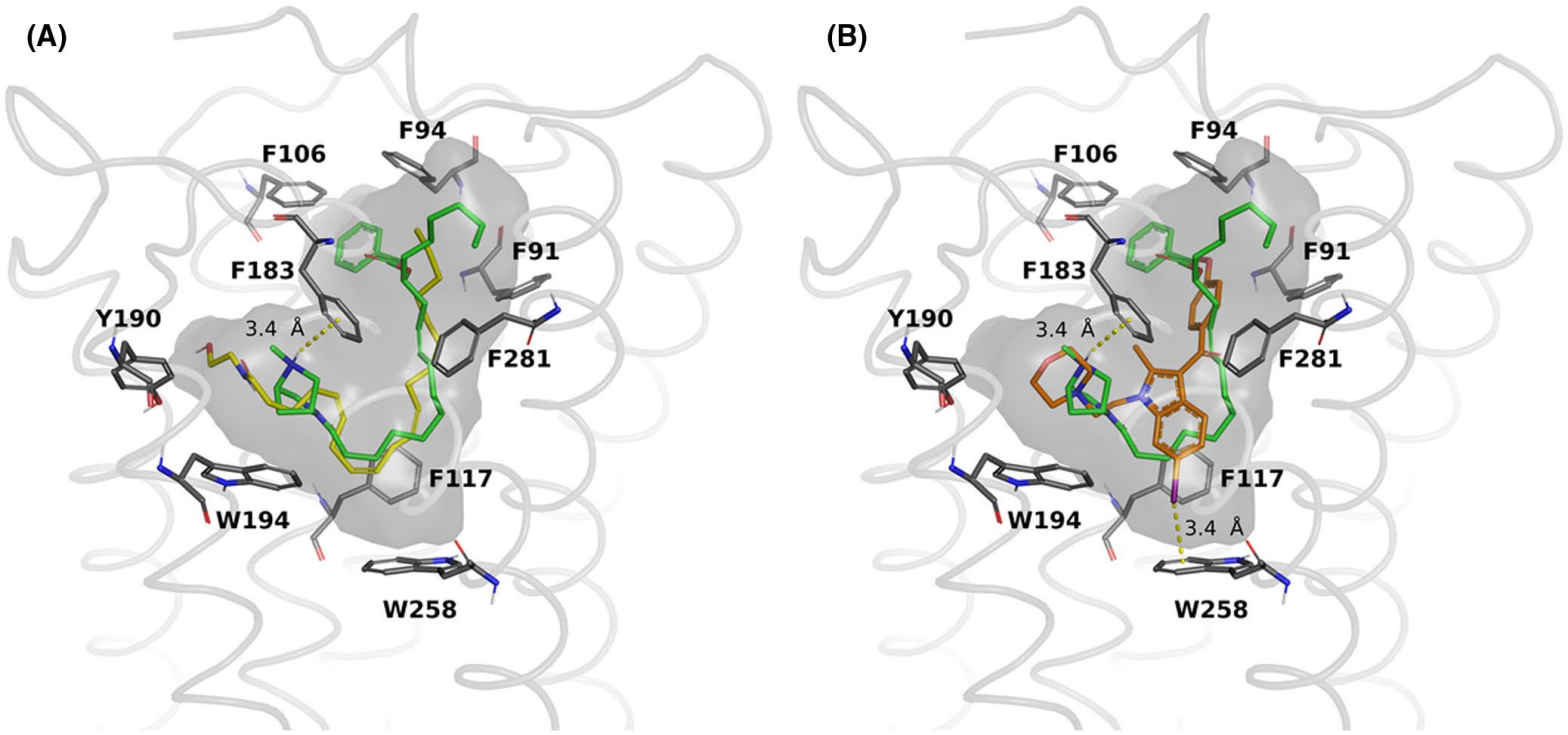
Binding mode comparison between PhAR‐DBH‐Me (green) and the endogenous agonist anandamide (yellow) **(A)**, or the synthetic antagonist of CB2 receptor, AM‐630 (orange) **(B),** in the binding pocket of CB2 receptor. The binding cavity is shown in grey, and relevant residues are included

On the CB2 receptor, Figure 3A highlights the similar binding poses between PhAR‐DBH‐Me and AEA with very similar docking scores (−8.3 vs −8.2 kcal/mol, respectively), whereas Figure 3B shows the comparison with respect to the antagonist AM630 (docking score of −7.2 kcal/mol). Similar to CB1, the antagonist hinders also the rotational interplay between the twin toggle residues, in this case F117/W258, while PhAR‐DBH‐Me and AEA are bound in a different section of the pocket, mainly through hydrophobic interactions.

**FIGURE 4:**
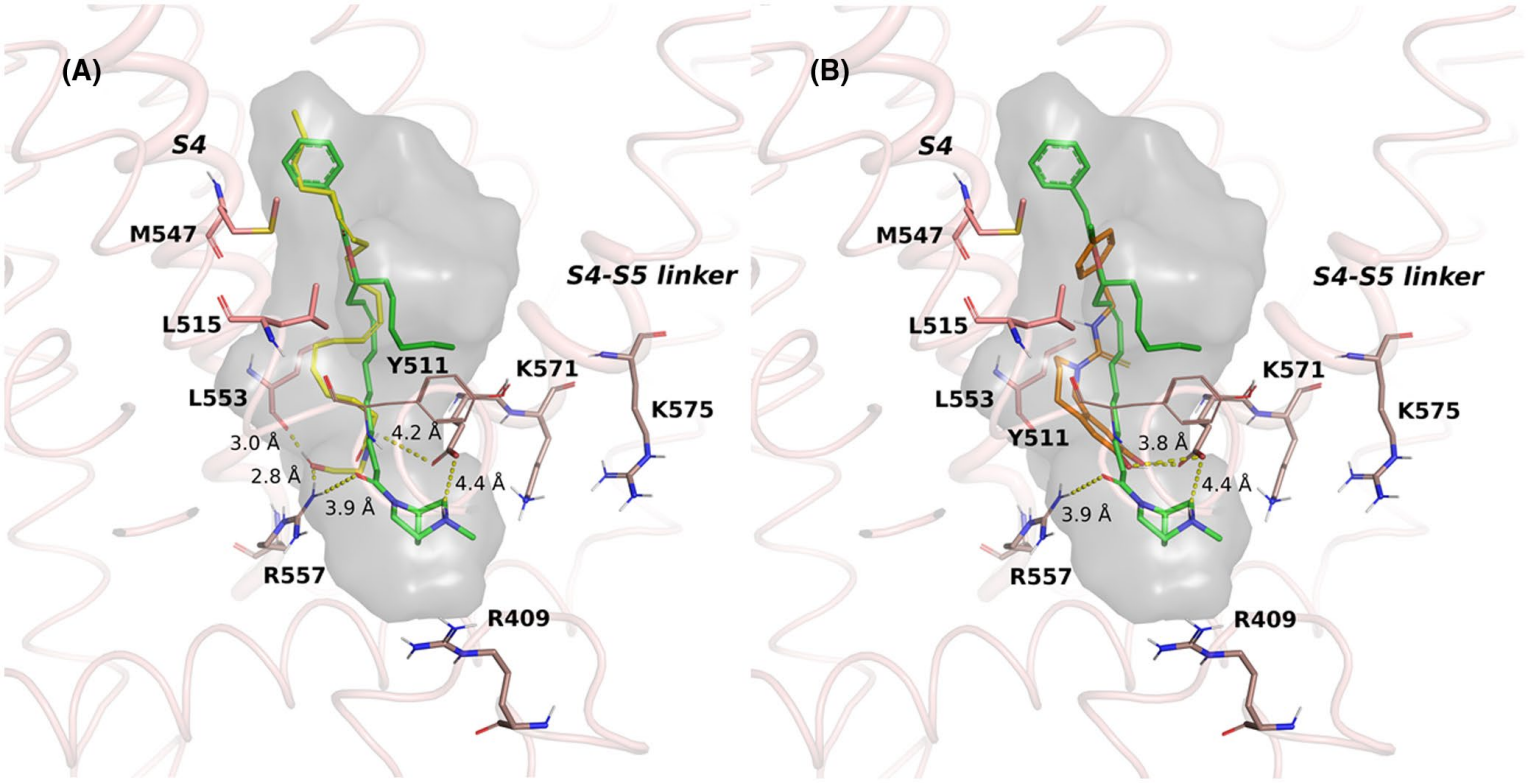
Binding mode comparison between PhAR‐DBH‐Me (green) and the endogenous agonist anandamide (yellow) **(A)**, or the synthetic antagonist of TRPV1 receptor, capsazepine (orange) **(B),** in the binding pocket of TRPV1 receptor. The binding cavity is shown in grey, and relevant residues are included

The TRPV1 receptor, which is an ion channel instead of a GPCR, shows similar interactions and binding modes between PhAR‐DBH‐Me and AEA, both interacting with key residues L553, R557, and E570 (Figure 4A). The docking scores (−6.9 and −6.4 kcal/mol, respectively) also support the possibility of PhAR‐DBH‐Me as a likely binder of TRPV1. In comparison, Figure 4B shows the binding mode of a known antagonist, capsazepine, interacting only with key residue E570, and thus blocking the interactions between R557/E570, fundamental for opening of the channel. The docking score of the antagonist is −7.1 kcal/mol.

Overall, computational studies strongly suggest that PhAR‐DBH‐Me is a likely binder for the three analyzed targets, also indicating an agonist character for the two GPCRs and an activator for the ion channel.

### 3.3 Intraperitoneal administration of AM‐251, AM‐630, and capsazepine prevents antiallodynic effect of PhAR‐DBH‐Me

**FIGURE 5:**
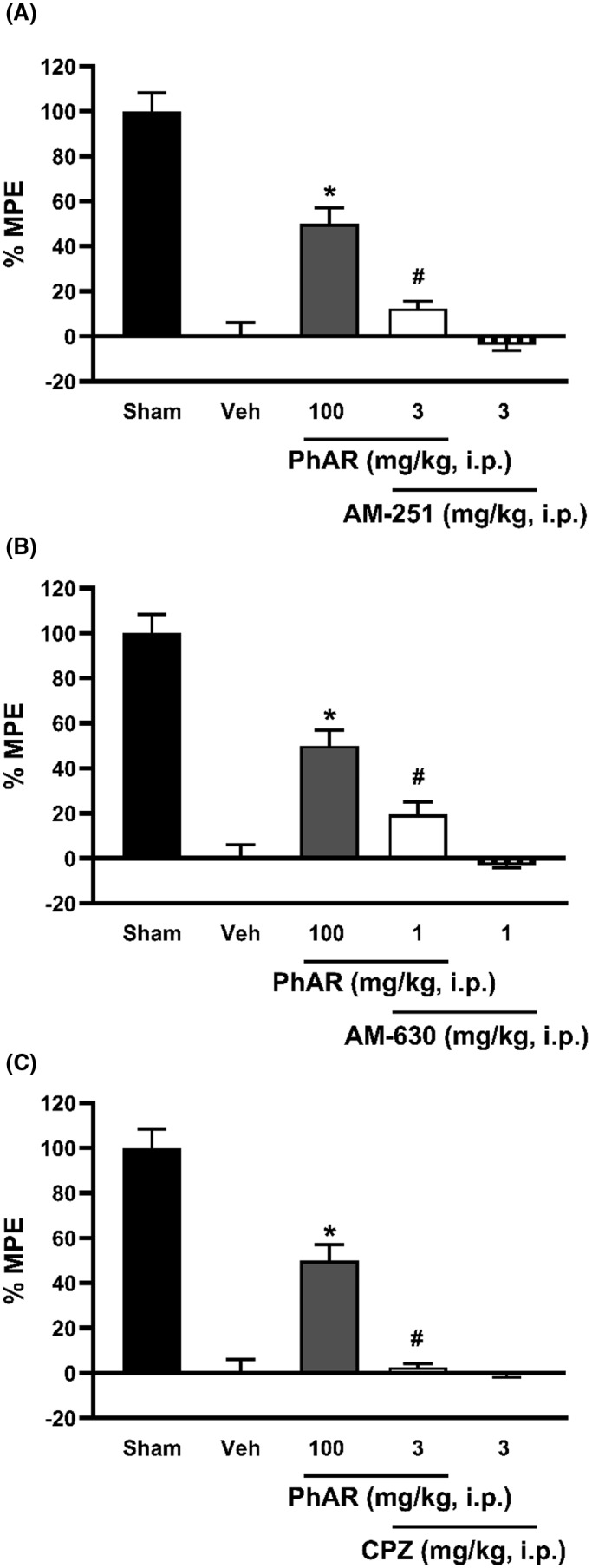
Effect of AM‐251 **(A)**, AM‐630 **(B)** and capsazepine **(C)** on antiallodynic effect induced by PhAR‐DBH‐Me (100 mg/kg, i.p.). Bars show the mean of the % Maximum Possible Effect (MPE) of 6 animals ± SEM. *P < .0001 vs SNL; *^#^P < *.0001 vs PhAR‐DBH‐Me, was determined by one‐way analysis of variance (ANOVA), followed by Tukey´s test

Concomitantly with computational studies, the intraperitoneal administration of AM‐251, a selective antagonist of CB1 (3 mg/kg) (Figure 5A) and AM‐630, a selective antagonist of CB2 receptor (1 mg/kg) (Figure 5B) prevented partially the antiallodynic effect of PhAR‐DBH‐Me (100 mg/kg) on L5/L6 spinal nerve ligated rats (*P ≤ *.0001). Interestingly, the administration of capsazepine, a selective antagonist of TRPV1 receptor (3 mg/kg) (Figure 5C) was a full preventer for PhAR‐DBH‐Me‐induced antiallodynic behavior on neuropathic rats. In contrast, the systemic administration of AM‐251, AM‐630, and capsazepine per se did not modify the antiallodynic behavior in rats. Taken together, in silico and in vivo data suggested that antiallodynic effect exerted by PhAR‐DBH‐Me involves the activation of CB1 and CB2 and a desensitization of TRPV1 receptors.

### 3.4 Capsaicin and PhAR‐DBH‐Me induces desensitization on vagal stimulation evoked esophagus contractions in rats

**FIGURE 6:**
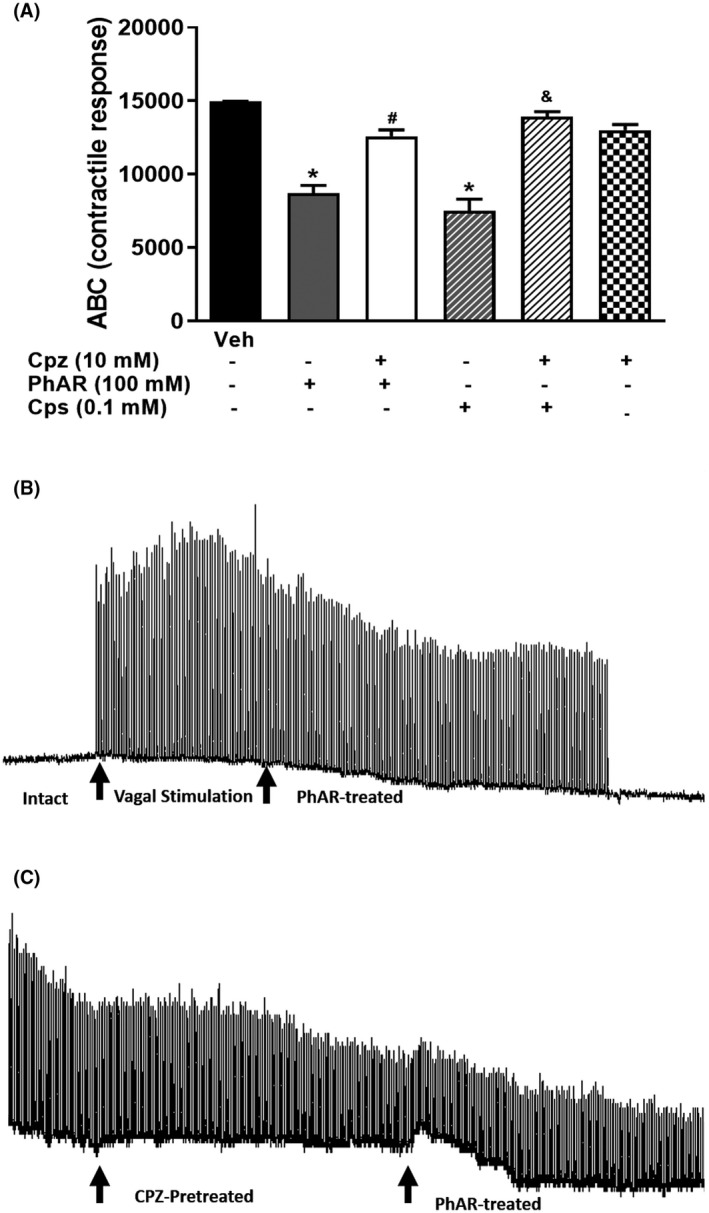
**(A)** Effect of the capzasepine (Cpz) on vagal nerve stimulation induced esophagus contractions in rat administrated with PhAR‐DBH‐ME. The bars shown the mean of the area under the curve of electrical stimulation of vagus nerve of 6 animals ± SEM. *P < .0001 vs veh (vehicle); *^#^P < *.0001 vs PhAR‐DBH‐Me, was determined by one‐way analysis of variance (ANOVA), followed by Tukey´s test. Cps, capsaicin (0.1 M); Cpz, capzazepine (10 mM); PhAR, PhAR‐DBH‐Me (100 mM). **(B)** Effect of the elevated electric stimulation on the vagus nerve on esophageal striated muscle contractility in the rat, followed by depressed response elicited by PhAR‐DBH‐ME and the **(C)** preventive effect of the Cpz on PhAR‐DBH‐Me‐ induced to decreased vagal nerve response. Electrical stimulations were applied to the vagus nerve using a square‐wave pulses (pulse duration 0.5 ms at intervals of 1 s) at intensity of 80 V. Black arrows indicate the different treatments applied to the vagus nerve

To test the pharmacological response of PhAR‐DBH‐Me on nervous tissue, we applied the electrical stimulation at vagus nerve. Results indicated that, vagal stimulation increased the contractile response on the striated muscle of the esophagus in the rats (Figure 6B). Moreover the single application of PhAR‐DBH‐ME (10 mM) to the vagus nerve, produced a significant decreased effect on the electrical response measured on the esophagus (Figure 6A and 6B), which in turn, was prevented by application of antagonist capsazepine, CPZ (10 nM) (Figure 6A and 6C). In similar form, capsaicin (0.1 M), decreased the electrical response, however, its effect was prevented by pretreatment with capsazepine (10 nM). Finally, capsazepine antagonist, did not produce effects on electrical response in the esophagus it self, induced by vagal electrical stimulation.

## 4 DISCUSSION

In this work, the intraperitoneal administration of PhAR‐DBH‐Me (3.2‐100 mg/kg) produced a significant antiallodynic effect on SNL‐ and cisplatin‐induced neuropathy in rats. This is the first report about the antiallodynic effect of PhAR‐DBH‐Me, which suggests its potential role in the treatment of neuropathic pain.

In silico studies strongly suggest an agonist/activator character for PhAR‐DBH‐Me. Although docking scores do not always show correlation when compared to binding affinity, they do give insights on compounds that are likely binders and its binding mode^30^, thus guiding on the target deconvolution tasks faced when understanding a mechanism of action.

The CB1 and CB2 receptors show large hydrophobic regions in their respective binding pockets, which allow a myriad of compounds to interact with them. For these two targets, the mechanism of action has been recently discussed^31^, suggesting that the blockage of the interplay of conserved residues F3.36/ W6.48 (according to the Ballesteros‐Weinstein nomenclature) is related to functional antagonism. For our systems, the docked poses show a high similarity, not only between the binding poses of the known agonist AEA, but also the docking scores, which suggest that the CB1 and CB2 receptor are likely targets of PhAR‐DBH‐Me. Moreover we observe both antagonists docking in different poses than AEA and PhAR‐DBH‐Me, effectively interacting with residues F200/W356 for CB1 and F117/W258 for CB2. Thus, this analysis gives a putative agonist role for PhAR‐DBH‐Me for both cannabinoid receptors.

For the TRPV1 ion channel there have been described two overlapping binding sites, one for vanilloid ligands and another one for phosphoinositide ligands^32^. These binding sites are found between the fourth transmembrane segment (S4) and a linker between segments 4 and 5 (S4‐S5 linker), where an interplay among residues Y511, R557 and E570 control the activation state of the receptor. For a more comprehensive work on the topology of TRPV1 ion channels, the reader is referred^33^.

In particular, when bound to a phosphatidylinositol (PTI) molecule, the sugar moiety can interact with the surrounding positively charged residues (R409, R557, K571, K575) and Y511 flexibility is constricted by the lateral chain of PTI, thus conserving the receptor into a closed state (Figure S1). When PTI gets displaced, the sugar pocket becomes void, allowing the interaction of R557 and E570 and the rotation of Y511. Ligands that favor the interaction of the S4 and S4‐S5 linker regions through its hydrophobic residues and the R557/E570 interaction open the ion channel, such as resiniferotoxin, while ligands that do not promote these interactions, such as capsazepine (CPZ), act as antagonists^34^.

For our systems, it can be observed that PhAR‐DBH‐Me and AEA locate their polar heads into the most internal section of the pocket, and bring together residues R557 and E570, without being locked by the surrounding positively charged residues, thus bringing together the S4 and S4‐S5 linker segments and favoring the activation of the channel. In comparison, CPZ interacts mostly only with E570, blocking the interaction with R557 and the opening of the channel. Therefore, these studies also suggest an activator role for PhAR‐DBH‐Me with respect to the TRPV1 ion channel.

Once realized the in silico screening, systemic administration of AM‐251 and AM‐630 partially prevented the antiallodynic effect of PhAR‐DBH‐Me, whereas that, the injection of capsazepine was a full preventer of the antiallodynic effect, which suggested that properties exerted by this novel compound requires activation of CB1, CB2, and TRPV1 receptors.

Our results matched with previously reported data^14^, relating the activation of CB1 receptor on PhAR‐DBH‐Me‐induced anti‐hyperalgesia in mice evaluated by the hot plate test^14^. Furthermore, our results extend the existing data, including the participation of CB2 and TRPV1 receptors as a part of the mechanism of action activated by PhAR‐DBH‐Me. Taken together, the in silico and in vivo evaluation of mechanism of action complete the characterization of PhAR‐DBH‐Me and indicate that this compound mimics anandamide actions, targeting on CB1, CB2, and TRPV1 receptors^35^, ^36^, ^37^. Neuropathic pain condition has a complex and multifactorial physiopathology. In this regard, decreased anandamide levels at peripheral, central, and supraspinal regions, have been related with development and maintenance of neuropathic pain^38^. Furthermore, a decrease in the enzymes related with synthesis of anandamide has been observed, as well as an increase with its degradation pathways at peripheral, central and supraspinal levels (Malek et al, 2014)^39^, ^40^, ^41^. Therefore, the endocannabinoid system has an important role between pain perception/analgesia.

Our data suggest that systemic administration of this synthetic compound can act as an analogue of anandamide. In line with this, PhAR‐DBH‐Me might be restoring anandamide deficiency induced by neuropathy and mimicking its actions. Our results indicate that PhAR‐DBH‐Me is a partial activator of CB1 and CB2 receptors, which coincides with the fact that anandamide is a partial agonist at the CB1 and CB2 receptors^42^, ^43^. Both receptors are coupled through G_i/o_ proteins^36^, and CB1 is located on presynaptic terminals, in DRG and dorsal horn neurons^44^, ^45^, ^46^, whereas CB2 is highly expressed on glial cells^47^, ^48^, ^49^. This cannabinoid receptor distribution explains the antiallodynic effect of synthetic cannabinoids, which in turn acts by inhibiting the hyperexcitability of sensory neurons and the releasing of neurotransmitters such as glutamate or Substance P (SP) that maintains the aberrant circuits of pain^38^, ^45^, ^50^. Moreover CB1 and CB2 receptors are expressed on brain stem^51^, ^52^ and the agonism of this cannabinoid receptors on thalamus and amygdala are related with nociceptive transmission and modification of the emotional component of pain^53^.

We also studied the participation of TRPV1, which is involved in nociception^54^, and more recently it has been suggested its modulation by cannabinoids^55^. In fact, anandamide was the first full endogenous agonist to TRPV1 receptor^35^, ^56^. Our study suggests that the antiallodynic effect of PhAR‐DBH‐Me is strongly mediated by TRPV1 receptor and is prevented by capsazepine. In line with our results, two previous studies reported that capsaicin‐induced antiallodynic effect is reversed by capsazepine^57^ (Rho et al, 2009).

Interestingly, the antinociceptive effects of anandamide in spinal cord and descending pathways have been related with the activation of TRPV1^13^, ^58^. Recent reports suggest that neuropathic pain increases TRPV1 expression, as well as the Ca^2+^/calmodulin‐dependent protein kinase II (CAMKII) and extracellular signal‐regulated kinase (ERK) phosphorylation. These data confirm that there is a up phosphorylation (sensitization) of TRPV1 receptors on neuropathic condition^59^. In line with this, one of the acute desensitization theories suggest that protein kinase C (PKC) phosphorylates or sensitizes TRPV1 receptors; this sensitization occurs when the receptor is binding to analogues as capsaicin. Then, when anandamide‐like analogues bind, there is a quick increase in the influx of Ca^+2^, resulting in a higher activation of Protein phosphatase 2B (calcineurin, PP2B), which in turn induces a dephosphorylation and consequent desensitization. This desensitization is maintained during the TRPV1‐anandamide binding and is responsible of the analgesic effect^33^. It is suggested that anandamide transforms the ligand‐gated TRPV1 channel to a dephosphorylation‐gated channel. This process can explain the PhAR‐DBH‐Me‐induced analgesic effect observed in our study.

Structurally speaking, anandamide is a N‐acyl amide, whereas PhAR‐DBH‐Me is a bicyclic amide. Both showed a similar affinity in docking studies, which supports the hypothesis that PhAR‐DBH‐Me might be functioning as analogue of anandamide. In support of our data, others AEA analogs such as N‐arachidonoyl‐dopamine (NADA), N‐oleoyldopamine (OLDA), N‐palmitoyl‐ and N‐stearoyl‐dopamine (PALDA and STEARDA) are structurally similar to both capsaicin and anandamide, and have shown activity on TRPV1 receptors to induce analgesia, which suggest its use for pharmacological management of neuropathic pain^60^, ^61^, ^62^. Finally, is interesting to emphasize that there is co‐expression of the cannabinoid/TRPV1 receptors on DRG^63^, midbrain dorsal periaqueductal gray^64^, and partially, in prefrontal cortex^65^. In addition, the dual activation of TRPV1 and CB1 receptors in presynaptic and postsynaptic neurons of dorsal horn has been demonstrated^66^, ^67^. Hence, bibliographic evidence supports and highlights the antinociceptive multi‐target nature of our synthetic compound.

Finally, in order to clarify the role of PhAR‐DBH‐Me, the pharmacological response of it was measure in presence of antagonist capzasepine. We employed an isolated organ assay, in which, the neural reflex of vagus is propulsed to contractile response of esophagus striated muscle, where its response can be recorded and measured. This assay is normally used to elucidate the desensitization of drugs as capsaicin produce on neural circuit^68^. The local administration of PhAR‐DBH‐Me to the vagus nerve, produced a decreased electrical response on the esophagus of rat and, its effect was similar to observed with capsaicin administration, which has been reported by other studies^67^, ^68^. This result suggested that capsaicin and PhAR‐DBH‐Me induced desensitization on vagal nerve which in turn, is observed as a decrease the contractile response on esophagus of the rat. Moreover the antagonist capzasepine prevents the desensitization effect exert by PhAR‐DBH‐Me and capsaicin. Together, molecular docking, nociceptive behavior in the rats, as well, the isolated organ assay suggested the PhAR‐DBH‐Me produce a TRPV1 desensitization, with is prevented by the selective TRPV1 antagonist, capsazepine. This experiment was exclusively designed to evaluate the pharmacological response of PhAR‐DBH‐Me as a part of a new synthetic drug, hence, the results supports the hypothesis that PhAR‐DBH‐Me produce antinociceptive effects through desensitization of TRPV1 receptors.

Taken together, our results indicate that antiallodynic effect of PhAR‐DBH‐ME is mediated by activation of cannabinoid and vanilloid receptors, suggesting that the nature of this compound acts as an analogue of the endocannabinoid/endovallinoid system for control of neuropathic pain.

## AUTHORSHIP CONTRIBUTIONS

Navarrete, Rocha‐Gonzalez, and Quiñonez‐Bastidas *participated in research design*. Quiñonez‐Bastidas, Palomino‐Hérnandez, and González‐Anduaga *conducted experiments*. Palomino‐Hérnandez, López‐Ortíz, and Regla *contributed new reagents or analytic tools*. Quiñonez‐Bastidas, Palomino‐Hérnandez, González‐Anduaga, Rocha‐González, Navarrete, López‐Ortíz, and Regla *performed data analysis*. Quiñonez‐Bastidas, Palomino‐Hernandez, Rocha‐Gonzalez, and Navarrete *wrote or contributed to the writing of the manuscript*.

## DISCLOSURE

None of the authors have any conflict to declare.

## Supporting information

Fig S1

## Data Availability

Please contact the corresponding author for additional data requests. ORCID Andrés Navarrete https://orcid.org/0000‐0002‐2858‐8710
