# Supplementary figures and images for: Antiallodynic effect of PhAR‐DBH‐Me involves cannabinoid and TRPV1 receptors
Source: Pharmacol Res Perspect. 2020 Sep 23;8(5):e00663. doi: 10.1002/prp2.663 (PMC7510332; doi:10.1002/prp2.663)

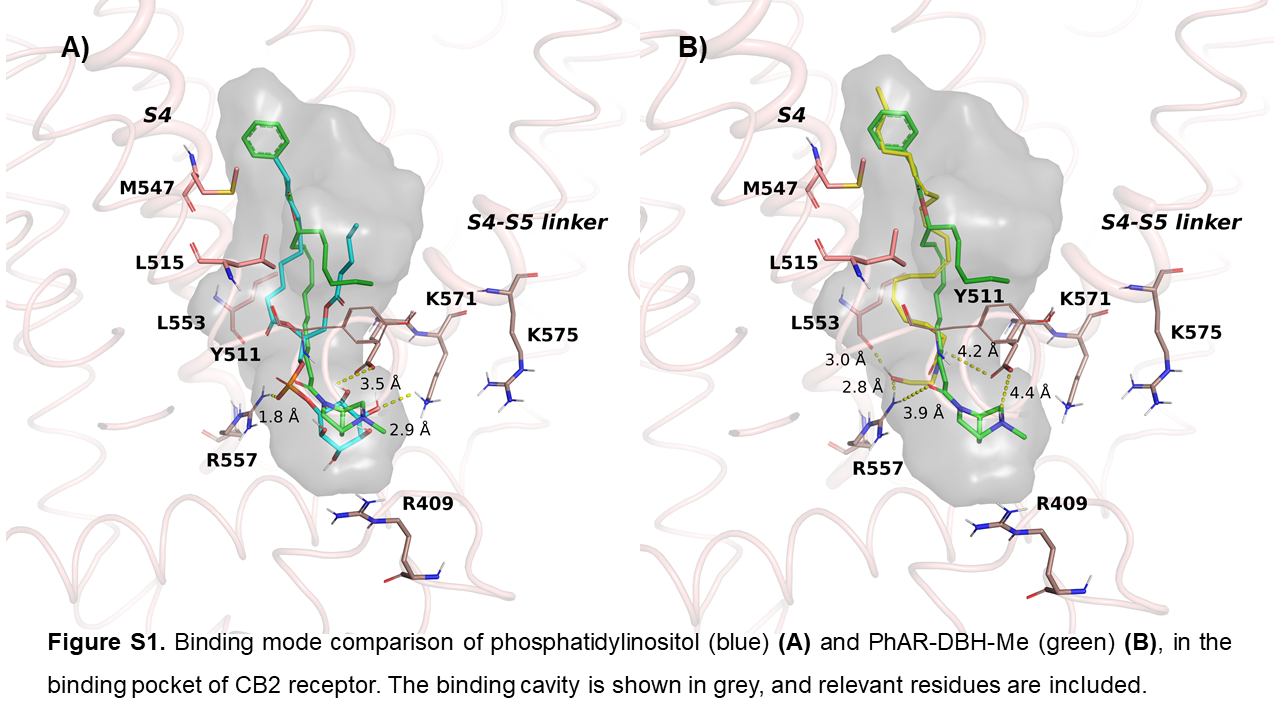

Supplement: Supplementary file 1 — Fig S1 [file PRP2-8-e00663-s001.tif]
